# How do health extension workers in Ethiopia allocate their time?

**DOI:** 10.1186/1478-4491-12-61

**Published:** 2014-10-14

**Authors:** Lindsay Mangham-Jefferies, Bereket Mathewos, Jeanne Russell, Abeba Bekele

**Affiliations:** Department of Global Health and Development, London School of Hygiene and Tropical Medicine, 15-17 Tavistock Place, London, WC1H 9SH UK; Department of Health and Nutrition, Save the Children, P. O. Box 387, Nefas Silk Lafto, Addis Ababa, Ethiopia; Department of Health and Nutrition, Save the Children, 2000 L St NW # 500, Washington, DC, 20036 USA

**Keywords:** Community health workers, Ethiopia, Health extension workers, Newborn health, Time and motion study

## Abstract

**Background:**

Governments are increasingly reliant on community health workers to undertake health promotion and provide essential curative care. In 2003, the Government of Ethiopia launched the Health Extension Programme and introduced a new cadre, health extension workers (HEWs), to improve access to care in rural communities. In 2013, to inform the government’s plans for HEWs to take on an enhanced role in community-based newborn care, a time and motion study was conducted to understand the range of HEW responsibilities and how they allocate their time across health and non-health activities.

**Methods:**

The study was administered in 69 rural kebeles in the Southern Nations Nationalities and People’s Region and Oromia Region that were intervention areas of a trial to evaluate a package of community-based interventions for newborns. Over 4 consecutive weeks, HEWs completed a diary and recorded all activities undertaken during each working day. HEWs were also surveyed to collect data on seasonal activities and details of the health post and kebele in which they work. The average proportion of productive time (excluding breaks) that HEWs spent on an activity, at a location, or with a recipient each week, was calculated.

**Results:**

The self-reported diary was completed by 131 HEWs. Over the course of a week, HEWs divided their time between the health post (51%) and the community (37%), with the remaining 11% of their time spent elsewhere. Curative health activities represented 16% of HEWs’ time each week and 43% of their time was spent on health promotion and prevention. The remaining time included travel, training and supervision, administration, and community meetings. HEWs spent the majority (70%) of their time with individuals, families, and community members.

**Conclusions:**

HEWs have wide-ranging responsibilities for community-based health promotion and curative care. Their workload is diverse and they spend time on activities relating to family health, disease prevention and control, hygiene and sanitation, as well as other community-based activities. Reproductive, maternal, newborn, and child health activities represent a major component of the HEW’s work and, as such, they can have a critically important role in improving the health outcomes of mothers and children in Ethiopia.

**Electronic supplementary material:**

The online version of this article (doi:10.1186/1478-4491-12-61) contains supplementary material, which is available to authorized users.

## Background

Health services in many low- and middle-income countries are constrained by a shortage of qualified health personnel [1]. To address this shortfall in human resources, governments are increasingly reliant on community health workers to undertake health promotion and to provide essential curative care. This role has been particularly important in rural areas, where access to care is often extremely limited [2]. Reproductive, maternal, newborn and child health promotion and selected services are frequently a major focus of the community health worker role, though many typically have a wide range of responsibilities [3]. Very little is known about how community health workers allocate their time, as relatively few time-motion studies are published and existing evidence is focused on facility-based health workers [4–8].

Over the past decade, the Government of Ethiopia has invested considerable resources in community-based preventive and curative services. The Federal Ministry of Health launched the Health Extension Programme in 2003 to help achieve universal access to primary health care and accelerate the country’s progress in meeting Millennium Development Goals for child mortality, maternal mortality, and in combating HIV/AIDS, malaria, and other diseases [1, 9]. The Health Extension Programme is now an established part of the health system and comprises 16 different health packages, organized under three themes: family health, disease prevention, and hygiene and environmental sanitation, with health education and communication as a cross-cutting theme (Table 1).

**Table 1:** **Sixteen health packages of the health extension programme**

Family health	Disease prevention and control	Hygiene and environmental sanitation
● Family planning	● HIV/AIDS and STIs	● Construction and maintenance of sanitary latrines
● Maternal, newborn, and child health	● Tuberculosis	● Solid and liquid waste disposal
● Nutrition	● Malaria	● Water supply safety measures
● Vaccination	● First aid	● Control of insects and rodents
		● Food hygiene and safety
		● Personal hygiene
		● Healthy home environment
● Health Education and Communication (cross-cutting)		

The Health Extension Programme introduced a new cadre of government health workers, known as health extension workers (HEWs), to deliver the programme at the community level in rural areas [1]. The government aims to recruit and train HEWs resident in the local community. HEWs are females who are at least 18 years old and have completed the 10^th^ grade of schooling. HEWs receive one year of training and are paid a government salary. More than 35,000 HEWs have been trained since 2003, and they work in more than 14,400 rural health posts across Ethiopia [9]. Each health post is built by the government, with significant inputs in the form of labour and materials from the local community, and serves an average population of 5,000 individuals. Most health posts are staffed by two HEWs, though up to four are deployed in larger communities. HEWs are supervised by nurses or environmental health professionals based at health centres in a nearby town. On average, a health centre oversees five health posts and together they comprise a Primary Health Care Unit (PHCU).

At the outset, the objectives of the Health Extension Programme were predominately health promotion and health prevention: to improve access to preventive essential health interventions at the household and village level; increase health awareness, knowledge, and skills; promote a healthy lifestyle; and improve the utilization of peripheral health services by bridging the gap between communities and health facilities [9]. A key strategy of the Health Extension Programme is to extend the reach of HEWs in partnership with community volunteer cadres. This approach is now being institutionalized and scaled up through the health system-wide Health Development Army (HDA). Development Team Leaders of the HDA each oversee up to six HDA network leaders in their communities [10]. Each network leader works with five neighbouring households to model and diffuse healthy practices. In an average kebele, there are approximately 30 Development Team Leaders and 200 HDA network leaders.

Over time, the Health Extension Programme has expanded services for curative care. In 2010, HEWs were given responsibility for integrated community case management (ICCM), which added the treatment of pneumonia and severe acute malnutrition to their existing responsibilities for management of malaria and diarrhoeal diseases in children aged 2 to 59 months.

Given the expanded range of HEW responsibilities and plans for HEWs to have an enhanced role in community-based newborn care, senior leadership in the Federal Ministry of Health expressed interest in learning more about how HEWs spend their working hours and, specifically, how much of their time is spent on preventive and promotive activities compared to curative activities. Moreover, to inform policy decisions on the introduction and scale-up of community-based newborn care, including community-based treatment of possible severe bacterial infections (PSBI) in the newborn by HEWs, the Federal Ministry of Health wanted to know how much time HEWs would be likely to spend identifying and treating sick newborns.

In this paper, we describe the range of HEW responsibilities and how they allocate their time across different health and health-associated activities. Specific objectives were to estimate the proportion of time HEWs spend per week on curative, preventive, and other activities, and also to estimate the time HEWs spend on infrequent and seasonal activities. This work complements previous research on the Ethiopian Health Extension Programme, which includes studies on its implementation [11], the knowledge and performance of HEWs [12], the role of HEWs in improving the utilization of maternal health services [13–15], and in tuberculosis case detection and treatment [16].

## Methods

### Study setting

The time and motion study was administered in rural kebeles in Sidama Zone of Southern Nations Nationalities and Peoples (SNNP) Region and in East Shoa and West Arsi Zones of Oromia Region. Kebeles are the smallest administrative structure in the government system and have an average population of 5,000. Each kebele has a health post, which is typically staffed by two HEWs, though two of the selected kebeles were staffed by three HEWs.

The selected kebeles were the intervention areas of a cluster-randomized controlled trial to evaluate the impact on mortality of a package of community-based interventions for newborns in Ethiopia (COMBINE), with and without community-based treatment of PSBI in the neonate by HEWs. The COMBINE trial was implemented by the Saving Newborn Lives program of Save the Children between 2008 and 2013 in partnership with the Federal Ministry of Health, Regional Health Bureaus, Zonal Health Offices, Woreda Health Offices, and John Snow Research and Training Institute, Inc.

In both arms of the trial, HEWs and community health volunteers were asked to work in partnership to conduct three home visits during pregnancy and five home visits during the first week of life. Establishing relative responsibilities for home visit timing was based on a careful analysis of projected levels of effort and other operational variables, with the understanding that HEWs alone would not be able to visit and counsel all mothers and assess newborns at the intervals required to achieve high coverage. Pregnancy home visits included promotion of antenatal care by a trained provider, birth preparedness, use of an insecticide-treated bed net, counselling on danger signs during pregnancy and delivery, and essential newborn care (clean cord care, early initiation of and exclusive breastfeeding, thermal care, and care-seeking for illness). Postnatal home visits included counselling for breastfeeding, clean cord and thermal care, counselling on danger signs in the postnatal care period (mother and newborn), assessing mothers and newborns for danger signs, and referral of mothers and newborns with danger signs to government facilities. In the intervention arm, HEWs were authorized to treat PSBI in the newborn with oral and injectable antibiotics when referral was not possible or acceptable to the family. COMBINE Project Officers provided supervision and support of HEWs and community health volunteers in partnership and collaboration with the Woreda Health Offices and health centres in both study arms.

### Study design

The time and motion study was designed to collect data on the amount of time HEWs allocated to different activities each week. The primary outcome measure was the proportion of time HEWs spend on curative activities per week. The study design included all 70 health posts in the intervention kebeles of the COMBINE trial: 34 in Oromia and 36 in SNNP, which together fall under the supervision of 11 PHCUs. Overall, there are 142 HEWs employed at these health posts, 68 HEWs in Oromia and 74 HEWs in SNNP.

The sample size calculation assumed all 142 HEWs working in 70 kebeles agreed to participate in the study. A survey sample of 142 HEWs keeping a diary over 4 weeks was calculated to estimate the primary outcome with a precision of ±6% (i.e., 44% to 56%), assuming a proportion of interest of 50%, intra-cluster correlation in HEWs’ time allocation between weeks of 0.3 and attrition of 15% [17]. The precision estimate is presumed to be conservative since 0.3 is a relatively high intra-cluster correlation and a proportion of interest of 50% gives the maximum expected range for primary outcome (if the observed proportion is higher or lower then greater precision would be achieved).

### Data collection

The principal mode of data collection was a diary that HEWs were asked to use to self-report all activities undertaken during each working day (Additional file 1). HEWs were asked to complete the diary on a daily basis for 4 consecutive weeks between 11 February and 10 March 2013 (which was 4 Yekatit to 1 Megabit 2005 in the Ethiopian calendar). To aid recording of activities in the diary, HEWs could choose from a menu of 28 health promotion and prevention activities, 12 curative health care activities, and 11 “other” activities. If needed, HEWs could describe additional activities (Additional file 1). HEWs were also asked to record where the activity took place and who received the activity (as applicable) from a pre-defined list.

HEWs were surveyed separately to collect supplementary data on selected characteristics of the HEWs, and selected details of the health post and the kebele in which they work. HEWs were also asked to recall the time spent on infrequent and seasonal activities in the past year, though we acknowledge this information may be subject to recall bias. The survey was completed by each HEW at the beginning of the data collection period. Field workers were trained to obtain written consent from HEWs, administer the survey, and orient each HEW in how to complete the diary using a pre-populated example. HEWs were assured that all information would be treated confidentially. Field workers collected completed diaries each week, and were available to answer questions about data collection.

The research instruments were developed specifically for the study and were piloted with HEWs from control sites of the COMBINE trial that did not participate in the time and motion study. Several formats for the diary were considered to ensure that the diary was quick and easy for the HEW to complete. The list of preventive, curative, and other activities were developed using descriptive references from the Health Extension Programme, as outlined in the Ministry of Health’s Implementation Plan. The list of activities was finalized following the pilot and after consultation with HEWs, staff at PHCUs, and COMBINE project staff. All activities were categorized as either i) preventive or promotive health activities, ii) curative health activities, iii) travel, or iv) other activities (Additional file 1). This categorization was necessary to ultimately answer the primary study question, and it is acknowledged that this may be an over-simplification. For example, a visit by a HEW in the first 48 hours following birth is preventive, but could include curative activities if treatment is indicated for neonatal sepsis or other conditions. In addition, the diary was designed to record the primary activity, and thus multi-tasking was not captured. The category of ‘other’ activities was included since HEWs spend time on administration and travel, take breaks, and may be involved in a wide range of activities in the local community.

### Statistical methods

Survey and diary data were entered into Microsoft Access 2007 (Microsoft Inc., Redmond, Washington, USA). Data were analyzed using Stata 12.1 (STATA Corporation, College Station, TX, USA). Descriptive statistics are used to summarize the findings from the HEW survey and the self-reported diary. The average number of minutes taken to travel to work and spent at work each day was calculated from the time the HEW reported she left home for work, arrived at work, and left work each day. All other activity results reflect the time allocation per week since each week HEWs are expected to allocate their time between the health post and the community. The results reflect the average proportion of productive time each week that a HEW spent on an activity, or at a location, or with a recipient. This was calculated by dividing the total number of hours each HEW recorded on an activity (or at a location, or with a recipient) by the total number of ‘productive’ hours that the HEW recorded in the diary for that week (where productive hours include all time recorded except breaks). Results are reported with the mean and the confidence interval around the mean, and allow for the complex study design in which HEWs recorded multiple weeks in the diary and whereby health posts were stratified by region.

### Ethics

Ethical approval for the COMBINE trial was obtained from the Ministry of Science and Technology (formerly the Ethiopian Science and Technology Agency) and London School of Hygiene and Tropical Medicine Ethics Committees. There was no monetary compensation for participants. HEWs were provided with a modest wristwatch to record time in their diaries, which they were allowed to keep at the study’s conclusion. Confidentiality of each participant was protected and information on individual participants was only available to the study team.

## Results

The self-reported diary was completed by 131 HEWs working in 69 health posts, and the supplementary survey was completed by 130 HEWs. In Oromia, one health post was closed and the two HEWs were not present for the study period, and 10 HEWs in Oromia were on annual leave for some or all of the data collection period. In SNNP, one HEW was on maternity leave during the data collection period.

### Attributes of HEWs

The attributes and working patterns of HEWs participating in the study are shown in Table 2. On average, HEWs were aged between 23 and 26 years old, had completed high school and 1 year of technical training, and had been working as a HEW for 5 to 6 years. Just over a third (35%) of HEWs reported they live in the kebele in which they work. HEWs reported it takes on average 50 minutes to travel to work, though there was a wide range. HEWs reported that in a normal working week, they would spend 3 days based at the health post, and 2 days working in the community. Approximately half of the HEWs reported they had worked on a Saturday or Sunday over the past 4 weeks, and on average they had worked 2 days over the past 4 weekends.

**Table 2:** **Health extension worker (HEW) attributes, working patterns, and kebeles**

	Oromia		SNNP		All	
	n = 57		n = 73		n = 130	
**HEW Attributes**	n	%	n	%	n	%
Highest level of education						
High school and 1 year of technical training	52	91%	66	90%	118	91%
High school and 2+ years of technical training	5	9%	7	9%	12	9%
Lives in the kebele in which work	18	32%	27	37%	45	35%
	Median	IQR	Median	IQR	Median	IQR
Age of HEW	24	(23–25)	25	(23–27)	24	(23–26)
Years has been an HEW	5	(4–6)	6	(5–7)	5	(5–6)
Minutes to walk from home to health post^a^	60	(28–90)	40	(25–75)	50	(25–90)
**Working Patterns**						
In a normal week (Monday–Friday), days spent working	Median	IQR	Median	IQR	Median	IQR
in the community	2	(2–3)	2	(2–2)	2	(2–3)
at the health post	2	(2–3)	3	(2–3)	3	(2–3)
In past 4 weeks, HEWs who have worked on	n	%	n	%	n	%
Saturday or Sunday	36	63%	31	42%	67	52%
Saturday	21	37%	31	42%	52	40%
Sunday	27	47%	23	32%	50	38%
If worked at a weekend in past 4 weeks, days worked (out of 8 days)	2	(1–2)	2	(2–2)	2	(1–2)
**Kebeles and Health Posts**	n = 33		n = 36		n = 69	
	Median	IQR	Median	IQR	Median	IQR
Development team leaders and community health volunteers active^b^	28	(25–35)	22.5	(12–20)	26	(20–31)
Model households active	110	(60–350)	337	(120–602)	187.5	(87–528)
Model households being trained	60	(50–150)	201	(108–330)	96	(58–230)
Estimated clients attending the health post per week	70	(30–90)	85	(47–200)	75	(35–125)

^a^Missing 11 from Oromia; ^b^Female community health volunteers were recruited under the COMBINE trial to provide pregnancy and postnatal home visits in their communities. IQR, Interquartile range; SNNP, Southern Nations Nationalities and Peoples.

Selected indicators of the HEWs’ working environment are also reported, including the number of development team leaders and community health volunteers HEWs manage, the number of households that have completed model household training and are currently active, those currently being trained, and model households that graduated and are currently practicing.

HEWs reported that, on average, 75 clients attend the health post per week. Health posts were well-equipped, with the majority having essential equipment and medicines in stock at the time of the survey (Additional file 2). HEWs were asked about the training they had received over the last 3 years and supervision they had received in the last 3 months (Table 3). In the last 3 years, more than 80% of HEWs had completed integrated refresher training, training in the management of PSBI, ICCM, and maternal, newborn, and child health. In addition, the majority (60% to 80%) of HEWs had received training on health management information systems, tuberculosis prevention and control, nutrition, and family planning. Approximately half of HEWs had received training in malaria prevention and control, HIV/AIDS and STIs, hygiene and environmental sanitation, immunization, and adolescent reproductive health. Only 18% of HEWs reported they had received training in first aid in the last 3 years.

**Table 3:** **Training and supervision received by Health Extension Workers (HEWs)**

	Oromia		SNNP		All	
	n = 57		n = 73		n = 130	
	n	%	n	%	n	%
**Training Received by HEWs** ^**a**^	**In past 3 years**		**In past 3 years**		**In past 3 years**	
Integrated refresher training	56	98%	67	92%	123	95%
Possible severe bacterial infections management	53	93%	66	90%	119	92%
Integrated community case management	56	98%	60	82%	116	89%
Maternal, newborn, and child health	52	91%	57	78%	109	84%
Health management information system	30	53%	72	99%	102	78%
Tuberculosis prevention and control	36	63%	62	85%	98	75%
Nutrition	41	72%	54	74%	95	73%
Family planning	36	63%	54	74%	90	69%
Malaria prevention and control	41	72%	31	42%	72	55%
HIV/AIDS and sexually transmitted infections	35	61%	31	42%	66	51%
Hygiene and environmental sanitation	32	56%	31	42%	63	48%
Immunization	32	56%	28	38%	60	46%
Adolescent reproductive health	24	42%	31	42%	55	42%
First aid	15	26%	9	12%	24	18%
**Supervision and Technical Support Received**	**In past 3 months**		**In past 3 months**		**In past 3 months**	
Any supervision or technical support^b^	54	95%	73	100%	127	98%
By Primary Health Care Unit (PHCU)^c^	53	93%	67	92%	120	92%
By Woreda Health Office (WoHO)^d^	43	75%	58	79%	101	78%
	Median	IQR	Median	IQR	Median	IQR
If supervised by PHCU, times in past 3 months	4.5	(2–8)	5	(3–8)	5	(2–8)
If supervised by WoHO, times in past 3 months	1	(1–2)	2	(1–2)	1	(1–2)

^a^Denominator includes do not know; ^b^Missing 3 from Oromia; ^c^Missing 1 from SNNP; ^d^Missing 1 from Oromia. IQR, Interquartile range; SNNP, Southern Nations Nationalities and Peoples.

Almost all HEWs reported they had received supervision in the last 3 months. The patterns of supervision were similar across the two regions. More than 90% had received supervision from the PHCU and, on average, HEWs had been supervised by the PHCU on five occasions. In addition, most HEWs had received one supervisory visit from the Woreda Health Office.

### Time allocation per week

Collectively, the 131 HEWs kept a diary for 496 weeks, which included 2,369 working days, and more than 20,000 activities. HEWs spend, on average, 50 minutes travelling to work and 7 hours and 49 minutes at work each day, based on the times they left home in the morning, arrived at work, and left work at the end of the day. HEWs logged an average of 5 hours and 27 minutes per day in the diary against specific activities, including breaks that averaged 29 minutes per day. Thus, there were, on average, 2 hours 22 minutes per day when HEWs were at work and did not record how they spent their time. On average HEWs recorded 4 hours and 58 minutes each day on ‘productive’ activities, which includes time logged against any activity except breaks.

The percentage of time HEWs spent each week on different activities, in different locations, and with different recipients was calculated using the amount of productive time each week as the denominator. On average, HEWs spent 43% of their time on health promotion and prevention, 16% on curative health care, 9% on work-related travel, and 32% on other activities (Table 4). Other activities include receiving training and supervision (9%), community meetings (8%), and administration (7%). HEWs spent just over half (51%) of their time at the health post, 37% in the community (with 15% in community outreach, 13% visiting households, and 9% on work-related travel), and 11% in other locations (which included meetings and training held at the PHCU). Almost half (44%) of the time on productive activities had a specific recipient, while a quarter (26%) was spent on activities that benefit the community as a whole. Thus, on average, HEWs spent 70% of productive time in contact with patients, families, or community members. This includes 11% of time with pregnant women, post-partum women, and their newborns; 15% with infants and children under 5 years of age; and 18% with adolescents, adults, and families. Non-contact time includes the time spent on administration, receiving training and supervision, and on work-related travel. The time allocation was similar across the two regions.

**Table 4:** Average proportion of productive time per week, by type of activity and by location*

	Oromia		SNNP		All	
	Mean	95% CI	Mean	95% CI	Mean	95% CI
**By type of activity:**						
Curative health activities	15.5%	12.4–18.6%	16.0%	13.9–18.1%	15.8%	14.0–17.6%
Preventive health activities	39.3%	35.6–43.0%	46.3%	41.9–50.6%	43.2%	40.3–46.2%
Work-related travel	10.7%	8.1–13.2%	7.8%	5.9–9.6%	9.0%	7.5–10.5%
Other activities	34.5%	30.8–38.2%	30.0%	26.1–33.8%	31.9%	29.2–34.7%
**By location:**						
At health post	49.5%	45.0–54.0%	52.9%	48.6–57.3%	51.4%	48.3–54.6%
In the community (outreach)	14.1%	11.2–17.0%	15.8%	12.2–19.3%	15.0%	12.7–17.4%
In the community (household)	15.8%	12.2–19.5%	11.6%	9.2–14.1%	13.4%	11.4–15.5%
Work-related travel	10.7%	8.1–13.3%	7.8%	5.9–9.6%	9.0%	7.5–10.5%
Other location	9.9%	7.3–12.6%	11.9%	8.7–15.2%	11.1%	8.9–13.2%
**By recipient:**						
In contact with patients, families or community	69.0%	65.3–72.6%	71.1%	66.7–75.5%	70.2%	67.3–73.1%
Pregnant women	8.5%	6.7–10.3%	6.0%	4.7–7.4%	7.1%	6.0–8.2%
Newborns and post-partum women (up to 1 month after birth)	4.1%	2.8–5.4%	4.3%	3.0–5.5%	4.2%	3.3–5.1%
Infants (aged 1 to 12 months)	6.3%	4.7–7.9%	8.1%	6.7–9.5%	7.3%	6.3–8.4%
Children (aged 1 to 5 years)	5.8%	4.1–7.5%	8.3%	6.8–9.8%	7.2%	6.1–8.3%
Adolescents (aged 6 to 18 years)	2.5%	1.5–3.5%	1.0%	0.5–1.5%	1.7%	1.2–2.2%
Adults (aged over 18 years)^a^	12.4%	10.4–14.4%	16.0%	13.4–18.6%	14.4%	12.8–16.1%
Families	1.0%	0.6–1.4%	2.7%	1.7–3.6%	2.0%	1.4–2.5%
Community	28.4%	24.0–32.7%	24.8%	21.428.1%	26.3%	23.7–29.0%
No recipient^b^	31.0%	27.4–34.7%	28.9%	24.5–33.3%	29.8%	26.9–32.7%

*Productive time is defined as the amount of time recorded in the diary each week on any activity except breaks. ^a^Excludes pregnant and post-partum women; ^b^Includes time spent on training, supervision, administration, and travel.

**Figure 1:**
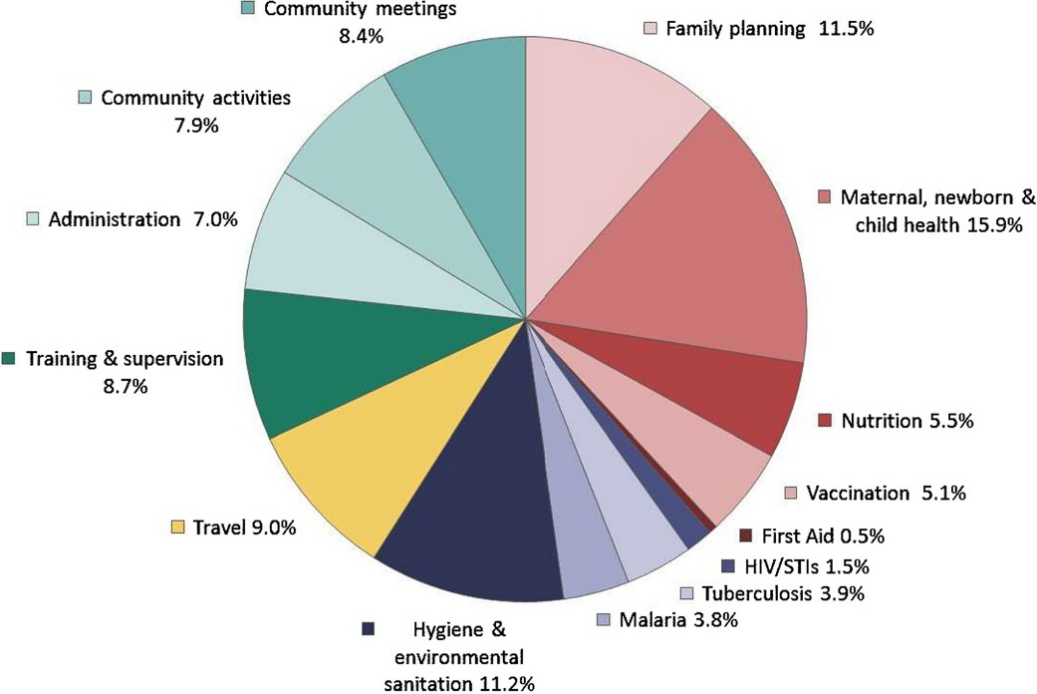
**Average weekly time allocation.**

In terms of the time spent on providing health care, family health was the largest component, with 38% of productive time allocated to family health services, 10% to disease prevention and control, and 11% to hygiene and environmental sanitation (the remainder was 9% on work-related travel and 32% on other activities) (Table 5). A further disaggregation shows HEWs spent just over a quarter (27%) of their time on family planning and maternal, newborn, and child health activities (Figure 1).

**Table 5:** **Average proportion of productive time per week by detailed activity**

Percentage of productive time per week on:	Oromia		SNNP		All	
	Mean	95% CI	Mean	95% CI	Mean	95% CI
**FAMILY HEALTH SERVICES**	32.5%	29.5–36.5%	42.5%	38.3–46.6%	38.1%	35.2–41.0%
Curative	8.8%	6.8–10.8%	12.7%	10.8–14.6%	11.0%	9.6–12.4%
Preventive	23.7%	20.8–26.6%	29.8%	26.4–33.1%	27.1%	24.9–29.4%
**Family planning** (all preventive)	8.2%	6.7–9.6%	14.2%	12.0–16.4%	11.5%	10.2–12.9%
**Maternal, newborn, and child health**	16.4%	13.3–19.5%	15.6%	13.5–17.6%	15.9%	14.2–17.0%
Curative	7.9%	6.0–9.9%	8.2%	6.7–9.8%	8.1%	6.9–9.3%
Preventive	8.5%	6.7–10.2%	7.3%	6.0–8.6%	7.8%	6.8–8.9%
**Vaccination** (all preventive)	4.9%	4.0–5.9%	5.2%	4.3–6.1%	5.1%	4.4–5.7%
**Nutrition**	3.0%	1.9–4.1%	7.5%	6.1–8.9%	5.5%	4.6–6.5%
Curative	0.9%	0.3–1.5%	4.5%	3.3–5.6%	2.9%	0.3–0.6%
Preventive	2.1%	1.2–3.0%	3.1%	2.2–3.9%	2.7%	2.2–3.6%
**DISEASE PREVENTION and CONTROL**	11.6%	9.2–14.1%	8.2%	6.6–9.7%	9.7%	8.3–11.0%
Curative	6.7%	4.8–8.6%	3.3%	2.2–4.4%	4.8%	3.7–5.8%
Preventive	4.9%	3.7–6.2%	4.8%	3.9–5.8%	4.9%	4.1–5.6%
**HIV and sexually transmitted infections**	2.1%	1.5–2.6%	1.1%	0.6–1.6%	1.5%	0.7–1.7%
Curative	0.5%	0.3–0.9%	0.5%	0.1–0.9%	0.5%	0.3–0.8%
Preventive	1.6%	1.1–1.9%	0.6%	0.3–0.9%	1.0%	0.8–1.3%
**Tuberculosis**	4.0%	2.9–5.2%	3.7%	2.9–4.5%	3.9%	3.2–4.5%
Curative	1.9%	1.0–2.8%	0.7%	0.2–1.1%	1.2%	0.7–1.7%
Preventive	2.1%	1.4–2.8%	3.0%	2.4–3.7%	2.7%	2.2–3.1%
**Malaria**	5.0%	3.3–6.6%	2.9%	1.8–3.9%	3.8%	2.9–4.7%
Curative	3.7%	2.2–5.2%	1.7%	0.8–2.6%	2.6%	1.7–3.4%
Preventive	1.3%	0.8–1.8%	1.2%	0.7–1.6%	1.2%	0.9–1.5%
**First aid** (all curative)	0.5%	0.3–0.8%	0.4%	0.2–0.6%	0.5%	0.3–0.6%
**HYGIENE and ENVIRONMENTAL SANITATION** (ALL PREVENTIVE)	10.7%	8.1–13.2%	11.7%	8.4–14.9%	11.2%	9.1–13.4%
**TRAVEL**	10.7%	8.1–13.2%	7.8%	5.9–9.6%	9.0%	7.5–10.5%
**OTHER**	34.5%	30.8–38.2%	30.0%	26.1–33.8%	31.9%	29.2–34.7%
Training and supervision	7.2%	5.2–9.2%	9.8%	6.6–13.0%	8.7%	6.7–10.7%
Administration	8.4%	7.0–9.7%	5.9%	4.8–7.0%	7.0%	6.0–7.7%
Community activities^a^	8.8%	7.0–10.6%	7.3%	5.3–9.3%	7.9%	6.6–9.3%
Community meetings	10.1%	7.8–12.5%	7.0%	5.4–8.6%	7.9%	6.6–9.3%

^a^Community activities include: meeting and training Health Development Army and community health volunteers, updating family folders, and updating Development Team Leaders listings. SNNP, Southern Nations Nationalities and Peoples.

Since the time and motion study was undertaken in the context of a trial on community-based newborn care, the allocation of time within maternal, newborn, and child health was of particular interest. ICCM and treating common childhood illnesses was the largest individual component and constituted on average 7.2% of a HEW’s time each week. HEWs also spent 3.9% of time on providing antenatal care at the health post, 1.8% on home visits to pregnant women, 0.4% supporting women in labour, 1.9% on postnatal visits (without community-based treatment), and 1.0% of their time treating PSBI in the neonate. The proportion of time HEWs spent on postnatal care and neonatal sepsis management is expected to be higher in the selected kebeles than elsewhere at the time due to the nature of the COMBINE study trial, though these activities represented a small percentage of HEWs’ time.

### Involvement in health campaigns and seasonal activities

HEWs were asked to recall whether they had been involved in health campaigns and selected seasonal and infrequent activities over the last 12 months (Table 6). These questions were intended to supplement information collected in the self-reported diary, as the diary was completed for 4 consecutive weeks and was not designed to capture activities that happen infrequently or only at specific times of the year.

**Table 6:** **Health extension worker (HEW) involvement in health campaigns and other infrequent activities**

CAMPAIGNS and INFREQUENT ACTIVITIES	Oromia				SNNP				All			
	n = 57				n = 73				n = 130			
	HEW has undertaken activity in past 12 months		If yes, number of days a HEW spent on activity in past 12 months		HEW has undertaken activity in past 12 months		If yes, number of days a HEW spent on activity in past 12 months		HEW has undertaken activity in past 12 months		If yes, number of days a HEW spent on activity in past 12 months	
	n	%	Median	IQR	n	%	Median	IQR	n	%	Median	IQR
Enhanced Outreach Strategy (deworming and/or vitamin A)	54	95%	5	(3–5)	69	95%	5	(3–5)	123	95%	4	(3–5)
Environmental Protection: Terracing (i.e., water shed management)	41	72%	15	(5–30)	31	42%	4	(3–15)	72	55%	9	(3–20)
Immunization campaign	39	68%	3	(3–5)	31	42%	3	(2–5)	70	54%	3	(2–5)
Given model household (including refresher) training	36	63%	15	(7–32)	31	42%	14	(3–22)	67	52%	15	(6–26)
Community-led total Sanitation campaign	28	49%	9.5	(3–25)	36	49%	7	(3–14)	64	49%	7	(3–14.5)
Family folder or development team leader listings/updating	30	53%	27	(15–45)	33	45%	13	(4.5–18)	63	48%	15	(8–30)
Malaria – indoor residual spraying campaign	25	44%	10.5	(2–17.5)	27	37%	7	(5–13)	52	40%	10	(6–15)
Malaria – environmental management	16	28%	10	(6–15)	22	30%	3	(2–5)	38	29%	5	(3–12)
Measles campaign	18	32%	3	(3–4)	10	14%	4	(3–5)	28	22%	3	(3–5)
Polio campaign	7	12%	4	(4–4.5)	1	1%	3	n/a	8	6%	4	(4–4)
Tax collection	23	40%	8	(3–20)	10	14%	1	(1–1.5)	33	25%	5	(2–10)
Maternity leave	7	12%	90	(90–90)	9	12%	90	(60–90)	16	12%	90	(60–90)
Other leave (including annual and sick)	25	44%	20	(15–24)	26	36%	18.5	(3–30)	51	39%	20	(10–30)

IQR, Interquartile range; SNNP, Southern Nations Nationalities and Peoples.

Almost all HEWs had been involved in the Enhanced Outreach Strategy, which distributes de-worming tablets and vitamin A supplements to children under 5 years old. Approximately half of the HEWs reported they had been involved in an immunization campaign, and also in campaigns to improve and protect the local environment, including community-led total sanitation initiatives and an agricultural program to promote terracing and improve watershed management.

About half of the HEWs had provided model household training in their community and had been involved in updating family folders (records of each family in the kebele) and development team leader listings in their kebeles. Each of these activities had taken on average 15 days per year. The malaria control campaign for indoor residual spraying was also relatively time intensive, taking an average of 10 days per year. A quarter of HEWs reported they had been involved in land use tax and agricultural income tax collection. Finally, almost 40% of HEWs indicated they had taken some annual, personal or sick leave over the past year, and 12% reported they had been on maternity leave for at least a portion of the last 12 months.

## Discussion

The Health Extension Programme was introduced to improve access to health promotion and care in rural Ethiopian communities. HEWs are responsible for providing family health services, improving disease prevention and control, and promoting improved hygiene and environmental sanitation, and are expected to divide their time between the health post and the community [18]. Curative health activities represented, on average, 16% of HEWs productive time, while promotive and preventive activities represented a larger proportion at 43%, on average. The remaining productive time includes work-related travel within the community (9%) and other activities (32%) such as administration, training and supervision, and community meetings.

Of the three pillars of the Health Extension Programme, family health services was the largest (38% of their time each week), with 10% of this time allocated to disease prevention and control, 11% to hygiene and sanitation, and 8% to community meetings. HEWs spend the majority (70%) of their time in contact with individuals, families and community members, which includes 26% with pregnant and postpartum women, newborns, infants, and children under 5 years old.

Over the course of a week, HEWs divide their time between the health post (51%) and the community (37%), with the remaining 11% of their time spent elsewhere. This practice is largely consistent with government policy, which advises HEWs to divide their time between the health post and the community. However, given the potential advantages of having HEWs living close to the health post, it was interesting to note that almost two thirds of HEWs reside outside the kebele in which they work.

This is the first published study to examine how HEWs in Ethiopia spend their time, and we believe this is the first, or at least one of the first, to report detailed information on how community health workers allocate their time. The peer-reviewed literature contains relatively few time and motion studies from low- and middle-income countries. The existing literature includes a study from Cameroon on the division of labour across health facility staff [4] and studies from Uganda on waiting time and patient flow at health facilities [5, 7], and on the amount of time clinician’s saved following the introduction of electronic medical records [6]. There is also a study from Ecuador that compared four methods for collecting data on clinician time at health facilities [8].

This study was designed to determine how HEWs allocated their time across a wide range of activities, and by asking HEWs to keep a diary and record each activity they completed over a 4-week period, we were able to obtain a large amount of detailed information with relatively limited resources. Other data collection methods are available and self-reported diaries can have limitations. For example, independent observation can improve the accuracy and consistency of data collection, but this method is resource intensive and can induce a Hawthorne effect as individuals may change their behaviour while under observation [19]. Self-administered diaries are usually considered more accurate than asking individuals to retrospectively estimate time use, given the potential for recall bias and for individuals to overestimate the amount of contact-time [8, 19]. The self-reported diary was piloted prior to data collection, which demonstrated HEWs were able and willing to complete the diary, and the field workers confirmed the HEWs had relatively few problems completing the diary. However, it is likely that some activities were under-reported since the amount of time HEWs recorded against activities was almost always less than the amount of time that would have elapsed between when they indicated starting and leaving work. This finding is consistent with the study from Ecuador that compared four different methods, as they found that health workers completing self-administered timesheets underestimated the amount of time spent on non-contact and non-productive activities when the timesheets were compared to independent observation [8].

Another methodological consideration was the timing and duration for data collection. A 4-week period was expected to provide reasonable detail on regular activities undertaken by HEWs, though we recognise it may not be sufficient to capture rare events, or to record the time spent on seasonal or infrequent activities. To our knowledge there were no specific seasonal or *ad hoc* activities underway during the data collection period, though it is not unusual for HEWs to be involved in specific health campaigns, under the direction of the PHCU and the Woreda Health Office. As it was unrealistic to expect HEWs maintain a self-reported diary over a prolonged period, we used interviews to collect information on seasonal and infrequent activities over the last 12 months, though we acknowledge this may be subject to recall bias.

This study was undertaken with HEWs who were participating in the COMBINE trial. As a result, the study health posts were better equipped as the trial supplemented essential medicines and supplies to avoid stock-outs, and the HEWs received frequent supervision from COMBINE staff as well as jointly with the PHCU and the Woreda Health Office when possible. This additional support may have had some influence on how HEWs allocate their time across the range of activities and may have resulted in greater emphasis on maternal, newborn, and child health and an increased amount of time spent receiving associated training and supervision. However, the amount of time HEWs spent on pregnancy and postnatal care home visits (including diagnosis and treatment of neonatal PSBI), which was the focus of the COMBINE trial, represented less than 3% of their time each week. This suggests extending HEW responsibilities to include home visits and the treatment of PSBI, one of the three major causes of death in newborns, is likely to have a relatively limited impact on the overall workload of HEWs, whilst having considerable potential to improve newborn survival [20]. The findings from this study were shared with the Federal Ministry of Health, and there was consensus that extending the Health Extension Programme to include management of PSBI in newborns would not unduly burden HEWs. However, some questions remain and additional research would be beneficial. For example, it would be useful to ascertain the extent to which HEWs have the capacity to take on additional responsibilities, how the evolving HDA influences the priorities and workload of HEWs, and how the HDA allocate their time.

## Conclusions

HEWs have wide ranging responsibilities for community-based health promotion and curative care in Ethiopia. Their workload is diverse, and over the course of a week they divide their time between activities relating to family health, disease prevention and control, hygiene and sanitation, and other activities. Reproductive, maternal, newborn, and child health activities represent a major component of the HEW’s work and, as such, they can have an important role in contributing to improved health outcomes of mothers, newborns, and children in Ethiopia.

## Electronic supplementary material

Additional file 1: **Self-reported diary and list of activities.** (DOCX 121 KB)

Additional file 2: **Equipment and supplies available at time of survey.** (DOC 58 KB)

## Abbreviations

COMBINE: Community-Based Interventions for Newborns in Ethiopia
HDA: Health development army
HEW: Health extension worker
ICCM: Integrated community case management
IQR: Interquartile range
PHCU: Primary health care unit
PSBI: Possible severe bacterial infection
SNNP: Southern Nations Nationalities and Peoples.
