# Supplementary material for: How do health extension workers in Ethiopia allocate their time?
Source: Hum Resour Health. 2014 Oct 14;12:61. doi: 10.1186/1478-4491-12-61 (PMC4209031; doi:10.1186/1478-4491-12-61)
Supplement: Supplementary file 1 — Additional file 1: Self-reported diary and list of activities. (DOCX 121 KB) [file 12960_2014_453_MOESM1_ESM.docx]

## Additional file 1: Self-reported diary & list of activities

| TOPIC | ACTIVITY CODE | ACTIVITY DESCRIPTION | CATEGORIZATION  (NOT SHOWN TO HEW)† |
| --- | --- | --- | --- |
| **MNCH** | 11 | Antenatal care | Promotive / Preventive |
|  | 12 | Pregnancy home visit | Promotive / Preventive |
|  | 13 | Delivery | Promotive / Preventive |
|  | 14 | Postnatal Care home visit (no possible severe bacterial infections (PSBI)) | Promotive / Preventive |
|  | 15 | Postnatal Care home visit (with PSBI treatment) | Curative |
|  | 16 | Treat PSBI (outside of Postnatal Care visit) | Curative |
|  | 17 | Treat diarrhoea | Curative |
|  | 18 | Treat pneumonia | Curative |
|  | 19 | Test and/or treat malaria (pregnant women and <5yrs) | Curative |
|  | 20 | Community case management | Curative |
| **First Aid** | 21 | First Aid (provide and/or refer) | Curative |
| **Nutrition** | 31 | Assess and treat severe acute malnutrition | Curative |
|  | 32 | Nutrition promotion campaign | Promotive / Preventive |
|  | 33 | Health Education on Nutrition | Promotive / Preventive |
| **Vaccination** | 41 | Vaccination | Promotive / Preventive |
|  | 42 | Health education on vaccination | Promotive / Preventive |
| **Family Planning** | 51 | Family planning services | Promotive / Preventive |
|  | 52 | Health Education on Family planning | Promotive / Preventive |
| **HIV/AIDS and/or STIs** | 61 | HIV/AIDS - Distribute condoms | Promotive / Preventive |
|  | 62 | Health Education on HIV/AIDS and/or Sexually Transmitted Infections | Promotive / Preventive |
|  | 63 | Voluntary Counselling and Testing | Curative |
|  | 64 | Assess for STIs | Curative |
| **Tuberculosis Prevention & Control** | 71 | Provide Directly Observed Therapy (DOT) for tuberculosis | Curative |
|  | 72 | Trace missing clients for Directly Observed Therapy | Promotive / Preventive |
|  | 73 | Active case finding for tuberculosis | Promotive / Preventive |
|  | 74 | Health Education on tuberculosis | Promotive / Preventive |
| **Malaria** | 81 | Distribute ITNs | Promotive / Preventive |
|  | 82 | IRS Campaign / Prophylaxis for high risk groups | Promotive / Preventive |
|  | 83 | Identify vector breeding sites / environmental management | Promotive / Preventive |
|  | 84 | Test and/or treat malaria (over 5yrs + adults) | Curative |
|  | 85 | Health Education on Malaria | Promotive / Preventive |
| **Hygiene &**  **Environmental Sanitation** | 91 | Construction, use and maintenance of sanitary latrines | Promotive / Preventive |
|  | 92 | Control of insects (except mosquitoes) and rodents | Promotive / Preventive |
|  | 93 | Food hygiene and safety | Promotive / Preventive |
|  | 94 | Healthy home environment | Promotive / Preventive |
|  | 95 | Personal hygiene | Promotive / Preventive |
|  | 96 | Solid and liquid waste disposal | Promotive / Preventive |
|  | 97 | Water supply safety measures | Promotive / Preventive |
|  | 98 | Health Education on Hygiene / Sanitation / Diarrhoea | Promotive / Preventive |
| **Other Activities** | 00 | Break (including lunch or tea) | Break |
|  | 01 | Travel for work purposes | Travel |
|  | 02 | Receiving training | Other |
|  | 03 | Receiving supervision | Other |
|  | 04 | Manage commodities and supplies | Other |
|  | 05 | Record keeping and reporting | Other |
|  | 06 | Family folder or Development Team Leader (DTL) Listings | Other |
|  | 07 | Meet with or train Health Development Army / Community Health Promoter | Other |
|  | 08 | Community Meeting - Health-related | Other |
|  | 09 | Community Meeting - Not health-related | Other |
|  | 10 | Other community-based activities | Other |

† This categorization was necessary for analysis though the categorization was not included in the list of activities given to HEWs.

| **RECIPIENT** | (SELECT ONE FROM THE LIST) | | |
| --- | --- | --- | --- |
| **Recipient** | 100 | None / Not relevant |  |
|  | 101 | Pregnant women |  |
|  | 102 | Post-partum mother | *(from birth to 6 weeks)* |
|  | 103 | Newborn | *(up to 1 month)* |
|  | 104 | Newborn + post-partum mother |  |
|  | 105 | Infant | *(1-12 months)* |
|  | 106 | Child | *(1-5 years)* |
|  | 107 | Adolescent | *(6-18 years)* |
|  | 108 | Woman | *(not pregnant or post-partum)* |
|  | 109 | Man |  |
|  | 110 | Family |  |
|  | 111 | Community |  |

| **LOCATION** | (SELECT ONE FROM THE LIST) | |
| --- | --- | --- |
| **Location** | 1 | Health Post |
|  | 2 | Community: Outreach |
|  | 3 | Community: With Household |
|  | 4 | Travel |
|  | 5 | Other (specify) |
