# Supplementary material for: How do health extension workers in Ethiopia allocate their time?
Source: Hum Resour Health. 2014 Oct 14;12:61. doi: 10.1186/1478-4491-12-61 (PMC4209031; doi:10.1186/1478-4491-12-61)
Supplement: Supplementary file 2 — Additional file 2: Equipment and supplies available at time of survey. (DOC 58 KB) [file 12960_2014_453_MOESM2_ESM.doc]

## Additional file 2: Equipment and supplies available at time of survey

|  | **Oromia** | | **SNNP** | | **All** | |
| --- | --- | --- | --- | --- | --- | --- |
|  | N=33 | | N=36 | | N=69 | |
| **Equipment and supplies at time of survey (observed or reported)** | n | % | n | % | n | % |
| Thermometer | 33 | 100% | 36 | 100% | 69 | 100% |
| Weighing scale | 32 | 97% | 36 | 100% | 68 | 99% |
| MUAC tape | 33 | 100% | 36 | 100% | 69 | 100% |
| Stopwatch | 24 | 73% | 30 | 83% | 54 | 78% |
| First Aid Kit | 17 | 52% | 20 | 56% | 37 | 54% |
| HIV Test Kit | 11 | 33% | 11 | 31% | 22 | 32% |
| Malaria RDTs | 26 | 79% | 21 | 58% | 47 | 68% |
| Coartem (first-line anti-malarial) | 26 | 79% | 17 | 47% | 43 | 62% |
| DOT (for confirmed TB patients) | 24 | 73% | 27 | 75% | 51 | 74% |
| Condoms | 33 | 100% | 36 | 100% | 69 | 100% |
| Contraceptive Pill | 33 | 100% | 32 | 89% | 65 | 94% |
| Depoprovera | 33 | 100% | 35 | 97% | 68 | 99% |
| Implanon | 19 | 58% | 33 | 92% | 52 | 75% |
| Folic Acid | 27 | 82% | 26 | 72% | 53 | 77% |
| Iron Supplements | 28 | 85% | 32 | 89% | 60 | 87% |
| Amoxicillin | 32 | 97% | 36 | 100% | 68 | 99% |
| Cotrimoxizole | 31 | 94% | 34 | 94% | 65 | 94% |
| Gentamicin | 33 | 100% | 36 | 100% | 69 | 100% |
| Vitamin A | 26 | 79% | 35 | 97% | 61 | 88% |
| Zinc | 30 | 91% | 7 | 19% | 37 | 54% |
| Oral rehydration salts | 29 | 88% | 34 | 94% | 63 | 91% |
| Paracetamol | 28 | 85% | 33 | 92% | 61 | 88% |
